# Linking environmental injustices in Detroit, MI to institutional racial segregation through historical federal redlining

**DOI:** 10.1038/s41370-022-00512-y

**Published:** 2022-12-21

**Authors:** Abas Shkembi, Lauren M. Smith, Richard L. Neitzel

**Affiliations:** Department of Environmental Health Sciences, University of Michigan, Ann Arbor, MI USA

**Keywords:** Environmental justice, Redlining, Structural racism, EJSCREEN, Noise, Air pollution

## Abstract

**Objectives:**

To identify the most pervasive environmental exposures driving environmental disparities today associated with historical redlining in Detroit.

**Methods:**

We overlaid Detroit’s 1939 Home Owners’ Loan Corporation (HOLC) shapefile from the Mapping Inequality project onto the EPA EJScreen and the DOT National Transportation Noise maps to analyze differences in current demographic and environmental indicators between historically redlined (D-grade) and non-redlined neighborhoods using simple linear regression and a boosted classification tree algorithm.

**Results:**

Historically redlined neighborhoods in Detroit experienced significantly higher environmental hazards than non-redlined neighborhoods in the form of 12.1% (95% CI: 7.2–17.1%) higher levels of diesel particulate matter (PM), 32.2% (95% CI: 3.3–69.3%) larger traffic volumes, and 65.7% (95% CI: 8.6–152.8%) higher exposure to hazardous road noise (L_EQ(24h)_ >70 dBA). Historically redlined neighborhoods were situated near 1.7-times (95% CI: 1.4–2.1) more hazardous waste sites and twice as many (95% CI: 1.5–2.7) risk management plan (RMP) sites than non-redlined neighborhoods. The lifetime cancer risk from inhalation of air toxics was 4.4% (95% CI: 2.9–6.6%) higher in historically redlined communities, and the risk of adverse respiratory health outcomes from air toxics was 3.9% (95% CI: 2.1–5.6%) higher. All factors considered together, among the environmental hazards considered, the most pervasive hazards in historically redlined communities are proximity to RMP sites, hazardous road noise, diesel PM, and cancer risk from air pollution.

**Conclusions:**

Historically redlined neighborhoods may have a disproportionately higher risk of developing cancer and adverse respiratory health outcomes from air toxics. Policies targeting air and noise pollution from transportation sources, particularly from sources of diesel exhaust, in historically redlined neighborhoods may ameliorate some of the impacts of structural environmental racism from historical redlining in Detroit.

## Introduction

In the wake of the Great Depression, the federal government sought to revitalize the economy by stabilizing a critical source of wealth—homes—for Americans. The Home Owners’ Loan Corporation (HOLC) was created in the New Deal to steady the housing market in major cities across the US by providing low-interest, long-term mortgage loans [1]. After the HOLC’s inception, its systemic racial and ethnic discrimination became evident and continues to impact urban centers across the US today.

**Fig. 1:**
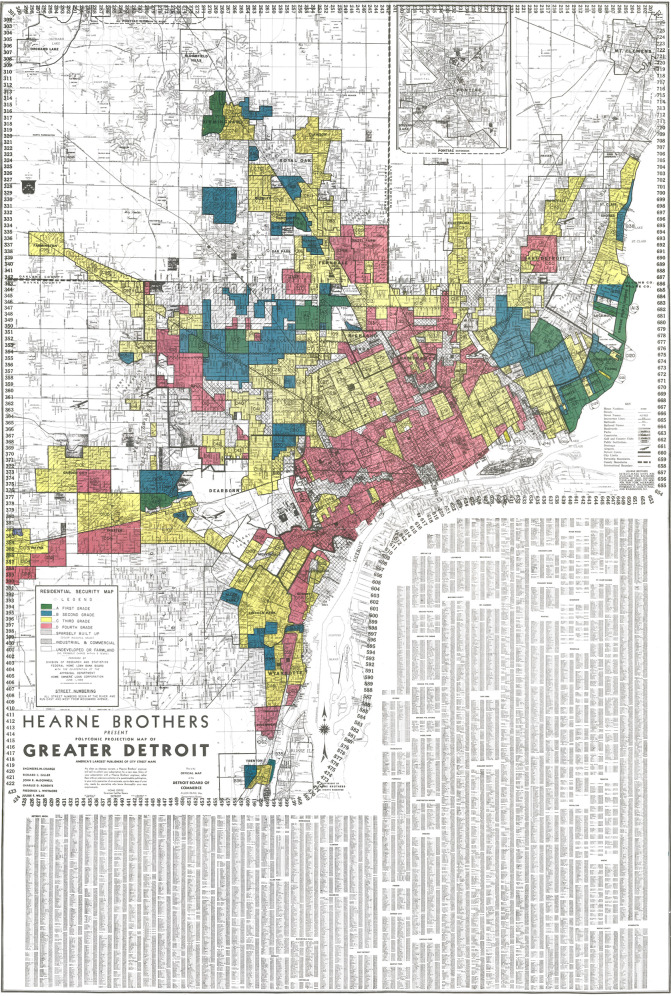
Scan of the 1939 Home Owners’ Loan Corporation (HOLC) map of the city of Detroit. Source: https://dsl.richmond.edu/panorama/redlining/#loc=5/39.1/-94.58&text=downloads.

The 1939 map of Detroit, Michigan (Fig. 1) is a colorful representation of the city, as imagined by the HOLC. Each residential neighborhood at the time was categorized into four grades based on perceived mortgage risk: A—“best”, in green; B—“still desirable”, in blue; C—“definitely declining”, in yellow; and D—“hazardous”, in red. The coloring of these maps may appear arbitrary; in the town of Inkster, southwest Detroit, a green-colored neighborhood (A15) rated “best”, is only blocks away from a red-colored “hazardous” neighborhood (D54). However, these colors were not arbitrary at all. Detailed remarks for D54 were simple and succinct: “almost entirely occupied by negroes”. Neighborhood A15, despite being near industrial zones and having “not very strong” property values, was noted to have “desirable home owners” and one of the most “desirable neighborhood[s]” in the city, with “negroes: 0%” and “foreign families: 0%”. Writing in his Underwriting Manual, a prominent real estate figure at the time explicitly described these racist motivations: “The infiltration of inharmonious racial groups… tend to lower the levels of land values and lessen the desirability of residential areas” [2]. This ideology was reinforced by the HOLC, with racial makeup being a significant determinant of the perceived mortgage risk of a neighborhood [3].

This phenomenon was not unique to Detroit. More than 200 US cities were subjected to the same racist, federal policy, which systematically denied mortgages to minorities (i.e., Blacks and immigrants/foreign-born) seeking homes in more wealthy/affluent neighborhoods due their “perceived mortgage risk” [3]. This policy segregated minority communities from white communities and helped shape racial housing segregation [4] by devaluing non-white neighborhoods [5]. Commonly referred to as “redlining”, the policy was a form of structural racism and discrimination that has and continues to have tremendous socioeconomic, environmental, and health implications today.

To begin to disentangle the legacy of historical redlining and provide evidence for remediation of its institutionalized impact across the US, we must consider the hypothesized mechanism that resulted in systemic environmental injustices. After a period of failed racial and ethnic integration following the Civil War, Jim Crow laws pervaded local, state, and federal policies to uphold racial segregation and marginalization of Black people. These spawned, among other things, local racial covenants which segregated whites from other communities [6]. Banks began denying mortgages to Blacks and/or immigrants who wished to integrate into white neighborhoods [6]. As such, race and ethnicity became a determinant of property value across the US, with the presence of Blacks and/or immigrants within a white neighborhood deemed disastrous to the neighborhood’s value [2]. Paradoxically, when it came time for the federal government to offer refinancing options in the 1930s, the government did not systematically deny refinancing options based on race and ethnicity [7], contrary to popular opinion regarding the HOLC’s involvement in the housing market. The federal government did, however, maintain the status quo of local redlining within each city [6, 7]. Further, the HOLC maps were created after all the loans were distributed [7]. Thus, the HOLC maps are not evidence of which neighborhoods were more likely to receive federal mortgage loans. Rather, they are reflections on how the federal government reinforced racial and ethnic segregation and the use of race as a significant determinant of property value [7]. HOLC’s 1930s formalization of “redlining” was how the federal government supported a diverse set of actors’ discriminatory beliefs on race and place which perpetuated racial and ethnic segregation across the US [8]. This provides evidence against de facto residential segregation, and shifts the ensuing impacts (e.g., environmental) onto the institutions themselves (i.e., de jure residential segregation) [5, 6]. As a result, the practice of redlining was systematic, pervasive, and formalized the evaluation of residential properties by race and ethnicity. With Black and immigrant communities institutionally segregated and having had adjacent neighborhoods intentionally zoned industrial and torn down through the development of highways and interstate freeways, they have been and continue to be “systematically targeted for the siting of noxious facilities”, and thus bear the brunt of environmental pollution in the US today [9–11].

Within the context of historical redlining, recent studies have shown associations between redlining and heat stress and higher intra-urban heat [12], less greenspace [13, 14], higher incidence of brownfield sites [14], oil and gas well siting [15], and air pollution [8, 16]. Poor health is a well-understood consequence of environmental exposures; redlining has also been associated with higher cardiovascular disease risk [17], asthma risk [18], preterm birth [19, 20], breast cancer mortality [21], and generally poorer mental and physical health [22–24]. In tandem with these recent redlining studies, research over the last several decades has produced a substantial body of literature that challenges the notion that racial/ethnic minorities (in particular, Blacks) themselves are to blame for unequal racial outcomes in regard to health, socioeconomic status, and environmental pollution [25]. They highlight that these unequal outcomes persist precisely due to laws entrenched in US society that have been designed for the purpose of discrimination (e.g., redlining), due to policies which have intentionally circumvented the rights of racial/ethnic minorities (e.g., industrial land zoning), and due to the discrimination of the past [25]. Our study adds to the body of redlining literature in the context of a highly racialized city—Detroit.

Detroit has had a long and well-known history of structural racism and discrimination through environmental injustices relating to mass industrialization (i.e., car manufacturing, steel mills, and oil refineries), housing discrimination (i.e., the “white flight” of post-1950s in suburban Detroit), and finally, deindustrialization. Previous work has characterized the substantial environmental racism occurring in the city, particularly to air pollution, proximity to landfill and superfund sites, Toxic Release Inventory site activity, and overall cumulative risk [26–28], situating Detroit as an important city to examine the local impact of historical redlining on environmental injustices. Environmental injustices in Detroit have been well-characterized; however, whether these place-based injustices may relate to racialized housing policies from the lens of historical redlining is still not evident. Further, recent studies have investigated the relation between redlining and one or a few environmental pollutants. For policymakers to remediate potential environmental injustices, they must examine a wide range of environmental exposures and target the pollutants which pervade historically redlined neighborhoods. The objectives of this paper were to: (1) examine associations between historical redlining and current-day environmental exposures, and; (2) identify the most pervasive environmental exposures driving environmental disparities from historical redlining in Detroit. In other words, Objective 2 was intended to identify environmental exposures that most characterize redlined neighborhoods compared to non-redlined neighborhoods in Detroit. Environmental exposures investigated in this study include those within the regulatory scope of the US Environmental Protection Agency (EPA), as well as an important yet often under-considered exposure, noise pollution, to empower policymakers in Detroit to remediate environmental racism that may have stemmed from one indicator of systematic residential segregation—historical redlining.

## Methods

We leveraged three secondary data sources. First, we accessed Detroit’s historical HOLC shapefile from the University of Richmond’s Mapping Inequality Project [29]. Second, we overlaid this shapefile onto a map of Michigan in the EPA EJScreen, a national tool containing metrics of environmental and demographic indicators at multiple spatial scales. The overlay approach allows one to calculate any metric in EJScreen tool within any boundary. In this case, our estimates inside the EJScreen tool were based on the boundaries of each neighborhood as defined by the HOLC map, thus generating HOLC-defined neighborhood, current-day pollution estimates. Lastly, we amassed 24-h traffic-related noise exposure point-estimates from the Department of Transportation (DOT) National Transportation Noise Map (NTNM) within HOLC-defined neighborhood boundaries as well. The pollutants chosen in this study represent pollutants within the regulatory scope of the EPA. Albeit not included in the EPA EJScreen, noise pollution is included in this study due to the widespread, but poorly appreciated, environmental harm it creates in terms of both hearing- and non-hearing- related health outcomes (e.g., cardiovascular disease, diabetes) and suggestions of environmental injustices across the nation [30]. Congress created an Office of Noise Control and Abatement (ONAC) within the EPA in 1972 and passed the Quiet Communities Act in 1978 to protect communities from harmful levels of noise pollution [31]. The elimination of ONAC’s budget in the early 1980s has prevented enforcement of noise abatement rules, or pursuit of advisory guideline limits; nevertheless, these rules still exist and could be enforced or encouraged. Furthermore, as of 2011, some protections over highway and construction noise exist via the Federal Highway Administration’s (FHWA) Procedures for Abatement of Highway Traffic Noise and Construction Noise [32]. As such, noise pollution from road sources was included in this study.

### 1930s HOLC map of Detroit

The shapefile from the Mapping Inequality Project [29] contains the geographical shape, neighborhood name, and HOLC grade of 238 neighborhoods in 1930s Detroit. Five neighborhoods could not be included due to boundary issues (see Appendix A and Table S1 of Supplementary Materials). Grade is a hierarchical factor from the aforementioned A to D ranking. Grade D represents structural racism and discrimination in Detroit, as a scan of the detailed appraiser remarks for D-grade neighborhoods in the city shows that the presence of any Black families or immigrants, regardless of socioeconomic status and housing quality, was sufficient for grading the neighborhood D, while the same cannot be said for C-grade neighborhoods, which were often reserved for more industrialized neighborhoods independent of the racial/ethnic makeup of the residents [29]. The distinction between D-grade and C-grade neighborhoods may be different in other cities, given the variability in local appraisers’ own biases.

### Environmental and demographic indicators

The EPA EJScreen version 1.0 [33] (https://ejscreen.epa.gov/mapper/ejscreen_v1/index.html; accessed May 13, 2021) was utilized to estimate current-day environmental and demographic indicators for each 1930s HOLC-defined neighborhood in Detroit using the shapefile. EJScreen contains 11 environmental indicators: air toxics cancer risk (lifetime cancer risk from inhalation of air toxics; count per 1 million; 2014); respiratory hazard index (ratio of exposure concentration to health-based reference concentration; 2014); diesel particulate matter (PM; µg/m^3^; 2014); PM_2.5_ (µg/m^3^; 2016); ozone (ppb; 2016); traffic proximity and volume (count of vehicles at major roads within 500 meters; 2017); lead paint (percent of housing units built pre-1960; 2013–2017); proximity (as a count of sites within 5 km) to Risk Management Plan (RMP) sites (2019), hazardous waste facilities (2019), Superfund sites (2019); and a wastewater discharge indicator (as a concentration of toxicity within 500 m; 2017). Six demographic indicators are provided from 2013–2017 American Community Survey estimates, expressed as population percentages: low-income, minority (people of color, specifically those who are not non-Hispanic, white-alone individuals), less than a high school degree, linguistically isolated, age under 5, and age over 64. Another demographic indicator, “D_Index_”, was created as the average percent of low-income and minority individuals in a neighborhood, as constructed by the EPA, to account for the intersection of low-income people of color [34]. The environmental and demographic indicators are as raw measurement averages, counts, and percentages.

### Road-based community noise

Road noise data from the DOT NTNM [35] (https://maps.dot.gov/BTS/NationalTransportationNoiseMap/) was generated by the DOT through the FHWA Traffic Noise Model 2.3 (TNM) [36]. By modeling various factors (e.g., roadway inventory, pavement material, daily traffic data), NTNM estimates 24-h road traffic-related noise (L_EQ[24h]_ in dBA). Using R v4.0.2 (Boston, MA, USA), we accessed coordinated DOT road noise estimates for Michigan. Noise estimates range from a 45 dBA to >100 dBA. These coordinates were extracted and merged with the 1930s HOLC shapefile of Detroit to assign each noise-estimate coordinate a neighborhood. Within the boundaries of each HOLC-defined neighborhood, we counted the number of road noise estimates that were >70 dBA, a level considered hazardous to hearing in a community setting [37], to determine the percentage of road noise by neighborhood as a “hazardous noise” environmental indicator. Figure S1 in Appendix B illustrates this process.

### Statistical analysis

All analyses were conducted in R v4.0.2. Boxplots by HOLC grade were used to present each demographic and environmental indicator. This is not a census tract-based analysis; we directly used the neighborhood boundaries as they existed in the 1939 HOLC map for Detroit. For the purpose of this analysis and the context of historical redlining in Detroit, we grouped neighborhoods graded A, B, and C together and considered them “non-redlined” to compare them to D-grade (or “redlined”) neighborhoods. We performed simple descriptive analysis to observe current-day demographic differences. We utilized simple linear regression to assess percent differences in “redlined” vs. “non-redlined” neighborhoods for all environmental indicators to accomplish objective 1, using a significance threshold of 0.05. Right-skewed environmental indicators were log-transformed. Lastly, we performed boosted classification tree (BCT) analysis—a supervised machine learning algorithm—to identify the most pervasive environmental exposures driving environmental disparities between redlined and non-redlined neighborhoods in Detroit (objective 2).

BCTs grow many decision trees using information from previously grown trees to minimize the error at each split of the tree. While the algorithm runs, each variable in the model is assigned a variable importance score (i.e., the variables selection count and contribution to model error reduction). Variables with higher importance are thus more indicative of the outcome and have more statistical importance to the model. For this analysis, the outcome is binary (historically redlined vs. non-redlined neighborhood), and environmental and demographic indicators were used as current-day indicators of whether a neighborhood was historically redlined. The findings from this analysis would indicate which environmental pollutants are most disproportionately present among redlined neighborhoods than non-redlined neighborhoods today, and thus markers of structural racism. Demographic indicators were included to adjust for confounding by demographics. Pearson’s correlations were used to drop highly correlated predictors (*r* > 0.7). Five hundred iterations were run using a Bernoulli distribution (logistic regression on a 0–1 outcome, 1 indicated a redlined neighborhood), with a shrinkage rate of 0.01 and a tree complexity of 2 (allowing 2-way variable interaction). The relationship between environmental indicators and probability of a historically redlined neighborhood were visualized with smoothed partial dependency plots.

Finally, to examine if disparities persisted even when comparing historically redlined neighborhoods to neighborhoods most similar to them (i.e., C-graded neighborhoods), all statistical analyses were repeated for comparison between D- and C-graded neighborhoods, excluding A- and B-graded neighborhoods,

## Results

### Demographics

Two hundred and thirty-three neighborhoods were included in this analysis. Redlined neighborhoods accounted for 26.2% (*N* = 61) of the neighborhoods. Figure S2 in Appendix B displays boxplots of each demographic indicator by HOLC grade. On average, the percent of people without a high school diploma was substantially higher among redlined (22.1%) than non-redlined neighborhoods (13.7%). Similarly, the percent of low-income people in redlined neighborhoods (61.6%) was substantially higher than those in non-redlined neighborhoods (43.7%) as was the percent of minorities (74.1% among redlined; 55.3% among non-redlined). No substantial differences were observed for the percent of the population less than age 5, greater than age 65, or linguistically isolated.

### Environmental exposures

**Fig. 2:**
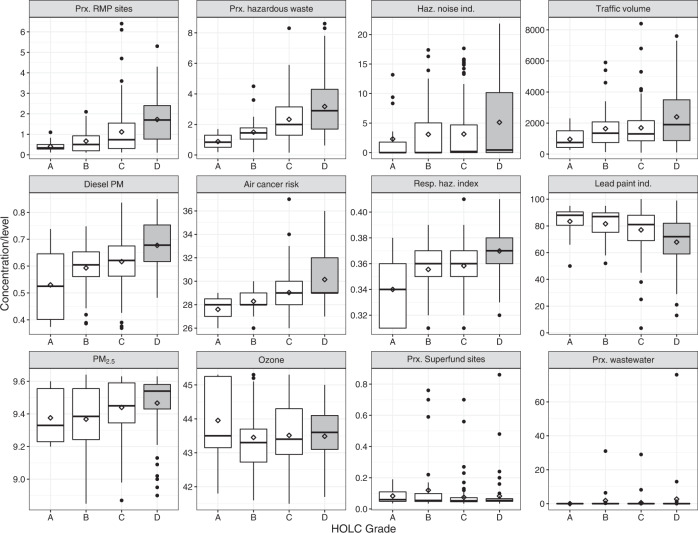
Boxplots of environmental indicators from the EPA EJScreen by 1930s HOLC grade. From left to right, top to bottom: proximity to Risk Management Plan (RMP) sites (count), proximity to hazardous waste sites (count), hazardous noise indicator (% road noise exposure>70 dBA), traffic volume (count), diesel particulate matter (PM, µg/m^3^), air cancer toxics risk (lifetime risk in 1 million), respiratory hazard index (ratio—unitless), lead paint indicator (% housing stock pre-1960s), PM_2.5_ (µg/m^3^), ozone (ppb), proximity to Superfund sites (count), and proximity to wastewater sites (count). Grade A signifies the “best” neighborhoods; grade B signifies “still desirable” neighborhoods; grade C signifies “definitely declining” neighborhoods; and grade D signifies “hazardous” neighborhoods. Redlined neighborhoods (grade D) are shaded gray; non-redlined neighborhoods (grades A, B, and C) are not shaded. Note that the *y*-axes are not consistent due to the different units of the environmental indicators. Diamond represents mean.

Figure 2 presents boxplots of each environmental indicator by HOLC grade. We observed that the lifetime cancer risk from inhalation of air toxics was 4.4% (95% CI: 2.9–6.6%) higher among redlined than non-redlined neighborhoods, as well as 3.9% (95% CI: 2.1–5.6%) higher for the respiratory hazard index among redlined neighborhoods. The average levels of diesel PM in the air were also 12.1% (95% CI: 7.2–17.1%) higher in redlined vs. non-redlined neighborhoods, and while no statistically significant difference was observed for levels of PM_2.5_, annual average levels were slightly elevated in redlined neighborhoods (0.5% higher; 95% CI: 0–1%). No statistically significant difference was observed for ozone levels. Traffic volume (32.2%; 95% CI: 3.3–69.3%) and percent of road noise >70 dBA (65.7%; 95% CI: 8.6–152.8%) were both significantly higher in redlined than non-redlined neighborhoods. Historically redlined neighborhoods had 1.7 (95% CI: 1.4–2.1) times more hazardous waste sites within 5 km and 2 (95% CI: 1.5–2.7) times more RMP sites within 5 km than non-redlined neighborhoods; they were not typically closer to Superfund sites and did not have statistically different counts of wastewater discharge sites than non-redlined neighborhoods. An inverse relationship was observed for the percent of houses built before 1960 (lead paint indicator); redlined neighborhoods have 13.6% (95% CI: 19.6–7.6%) less pre-1960 homes than non-redlined neighborhoods.

### Boosted classification trees

Percent of road noise exposure >70 dBA and traffic volume (*r* = 0.72), and diesel PM and respiratory hazard index (*r* = 0.87) were highly correlated. Therefore, traffic volume and respiratory hazard index were dropped for BCT analysis. Among the demographic indicators, high correlations were observed between percent people of color and percent low-income (*r* = 0.73), and percent low-income and percent without high school diploma (*r* = 0.79). The *D*_Index_ score was utilized instead since it was not strongly correlated (maximum *r* = 0.64) with any variables. To remain consistent with our objective of identifying the most predictive and negative environmental hazards associated with redlining, lead paint indicator (inverse relationship with redlining) was dropped. In total, five demographic and nine environmental indicators were included and the randomness threshold was set to 100/14 (7.14%) for the model.

**Fig. 3:**
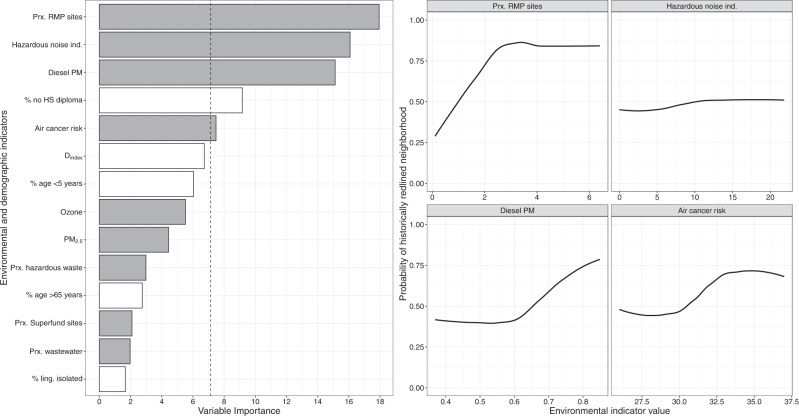
Results of the full boosted classification tree analysis. The left panel displays the relative variable importance of environmental and demographic indicators strongly associated with a redlined neighborhood compared to a non-redlined neighborhood. Predictors that exceed the dashed line (i.e., randomness threshold of 100/14, or 7.14%) are considered important factors that are most indicative of an area having been redlined. Gray bars are environmental indicators; white bars are demographic indicators. The right panel displays the partial dependency plots for the four environmental indicators above the randomness threshold in order of decreasing relative importance (from left to right, top to bottom): proximity to risk management plan sites (count); hazardous noise indicator (% road noise exposure >70 dBA); diesel particulate matter (PM, µg/m^3^); and air cancer toxics risk (risk in 1 million). The black line signifies the smoothed partial dependency plot.

Four environmental indicators and one demographic indicator exceeded the randomness threshold (Fig. 3—left panel). In order of decreasing variable importance, these important predictors were proximity to RMP sites (variable importance of 17.9%), percent of road noise >70 dBA (16.1%), diesel PM (15.2%), percent without a high school diploma (9.2%), and air toxics cancer risk (7.5%). The relationship between these important predictors and the likelihood of a neighborhood being historically redlined is shown in the right panel of Fig. 3. Generally, each predictor had a positive, sigmoidal relationship with the outcome, indicating that higher levels of each of these variables was significantly associated with higher probability of a neighborhood being historically redlined.

### Comparison of D- and C-graded neighborhoods

Table S2 in Appendix B presents the percent difference in environmental indicators between D- and C-graded neighborhoods and compared to differences between D- and A-C-graded neighborhoods. Overall, the percent differences between D- and C-graded neighborhoods were similar, although slightly smaller (i.e., toward the null), when compared to A-C-graded neighborhoods. Proximity to hazardous waste and RMP sites, the lifetime cancer risk from inhalation of air toxics, the respiratory hazard index, percent of road noise >70 dBA, and diesel PM all were significantly higher than C-graded neighborhoods. Only the volume of traffic was no longer significantly higher, although the difference remained positive.

The BCT model, comparing D- to C-graded neighborhoods only, provided similar results to the BCT model comparing D- to A-C-graded neighborhoods. Three environmental indicators exceeded the randomness threshold: proximity to RMP sites (variable importance 16.4%), diesel PM (15.9%), and percent of road noise >70 dBA (14.2%). However, the air toxics cancer risk no longer exceeded the randomness threshold and was the seventh most important variable in characterizing D-graded neighborhoods when compared to C-graded neighborhoods. Similarly, the percent without a high school diploma no longer exceeded the randomness threshold, but the *D*_Index_ score (average percent of low-income minorities) did with a variable importance of 7.9%. As indicated by the partial dependency plots, increasing values of each important variable still indicated higher probability of a neighborhood being historically redlined (Fig. S3 in Appendix B).

## Discussion

This study on the association between historical redlining and environmental hazards showed that there is a significant and continued disproportionate burden on segments of the population in Detroit that may be related to or stem from these institutional policies. Demographically today, redlined areas tended to have higher proportions of individuals that are low-income, without a high school diploma, or are from a minority population. These historically redlined neighborhoods experience significantly higher environmental hazards than non-redlined neighborhoods in the form of diesel PM, traffic volumes, hazardous road noise, cancer risk from air pollution, and are closer to hazardous waste and RMP sites. When these factors were considered collectively, the most pervasive environmental factors among neighborhoods historically redlined in Detroit are the proximity to RMP sites, hazardous road noise, diesel PM, and air toxics cancer risk.

Detroit has been included in nationwide analyses looking at the association between redlining and the environment or health outcomes [38–40] but few studies have looked at redlining specifically in Detroit. One study found that redlining may have worsened the impact on foreclosures from the Great Recession and self-reported health [41], while another found redlining not to be associated with superfund or landfill location from 1970 to 1990 [27]. White et al. showed that D-grade neighborhoods in Detroit had less high school graduates (consistent with our findings), more people without health insurance, less annual checkups, and higher poverty, but not always the highest prevalence of adverse health outcomes such as chronic heart disease, diabetes, and obesity [39]. Otherwise, studies on redlining have occurred in other cities. Across eight Californian cities, Nardone et al. similarly found that grade D neighborhoods had the highest levels of diesel PM, but not PM_2.5_ [18]. A nationwide study from Nardone et al. found that redlining was also systemically associated with less greenspace in a city, further exacerbating air quality issues [13] and one from Namin et al. showed worse air cancer toxics risk and respiratory hazard index among grade D [38]. While no other studies have examined proximity of RMP sites and noise pollution in the context of redlining, recently Krieger et al. linked redlining in Massachusetts to breast and lung cancer, thus supporting our observation of elevated air toxics cancer risk in Detroit [42].

Although not the most pervasive environmental pollutant, proximity to hazardous waste sites was associated with redlining in Detroit. This finding reflects some of the pioneering environmental justice research in the US, which identified the common practice of siting hazardous waste facilities in or near minority communities [10, 43]. On the other hand, one environmental indicator finding in Detroit may seem counter-intuitive—the lead paint indicator. This indicator is actually a measure of housing stock age (% of housing units built before 1960), and as such is a crude proxy for when lead paint use was discontinued. Due to urban blight in Detroit, thousands of historic homes have been razed, with low-income Black and immigrant communities specifically targeted [44], potentially explaining why redlined neighborhoods had a lower % of housing units pre-1960. This indicator is a poor marker of lead exposure within Detroit, and was not included in BCT analysis. However, it may pose a substantial threat in other cities across the US.

Disproportionately higher environmental exposures persist among historically redlined neighborhoods when compared to C-graded neighborhoods only. A- or B-graded neighborhoods were more likely to be dissimilar to D-graded at the time of HOLC appraisal, suggesting that differences between A/B- and D-graded neighborhoods may be attributable to other aspects of structural racism or social stratification unrelated to historical redlining [8]. Since C-graded neighborhoods in Detroit were often graded as such due to heavy industrialization and would be expected to have high levels of pollution, higher disproportionate environmental pollution today in historically redlined neighborhoods relative to C-graded neighborhoods may suggest intentional polluting of D-graded neighborhoods, or environmental racism. This notion is supported by the illustrated variable importance of the average percent of low-income minorities, *D*_Index_, in the BCT model as the demographic that characterizes historically redlined neighborhoods rather than C-graded neighborhoods today.

### Strengths and limitations

Environmental pollutants were limited to indicators within the EPA EJScreen and the DOT NTNM; other exposures, such as heavy metal and pesticide pollution, may alter the findings of the BCT analysis. However, the indicators chosen in this study represent pollutants within the regulatory scope of the EPA, which provides a legal avenue should the city of Detroit pursue remediation to overcome the environmental racism stemming from historical residential segregation. Similarly, most of the environmental indicators investigated came from the EPA EJScreen data, which generates point estimates of pollution levels and has measurement error associated with the data. These limitations should not come at the expense of action from policymakers, and our study reflects data that the EPA utilizes when assessing community exposures and risks. Unique characteristics of Detroit and nuances to redlining by city mean these findings may not be generalizable, and more research is needed in US urban centers with attention brought to each city’s historical context of land-use decisions and environmental injustices. We plan to investigate the heterogeneity of associations between redlining and environmental factors among all cities graded by HOLC, with the caveat that a larger scale, nationwide analysis diminishes the potential advantage of interpreting the findings in the context of each city’s history of environmental racism and would not account for the large variability in the appraisal process across cities conducted by city-specific appraisers. The ecological design of this study and the use of modeled data spanning several years (2013–2018) limits causal inference; future research should build cohorts to investigate environmental injustices and potentially associated health disparities at the individual level.

This study has important strengths worth highlighting. BCT provides a novel way to rank the importance among variables and allows for non-linear and interactive effects between variables of interest, reducing the likelihood of observing spurious relationships or overlooking an important relationship. The BCT model allows for direct translation of findings into actionable items for various stakeholders from community members to government officials. In combination with information on the magnitude of health risks linked to various environmental pollutants, as well as economic feasibility of environmental interventions, the BCT model provides valuable information on environmental efforts to remediate environmental injustice. This paper also investigated the role of noise—an often-overlooked, ubiquitous exposure with substantial health implications—as an environmental justice issue, and reiterates the importance of examining noise pollution in future studies of environmental disparities. This study used 1939 HOLC-defined neighborhoods to assess demographic and environmental aspects today. This approach eliminates the mess of accounting for changing Census Tract boundaries overtime and allows for a more specific assessment among the areas that were directly impacted by a discriminatory policy nearly 100 years ago—a place-based approach to understanding environmental injustice. Lastly, a focus on associations between historical redlining and environmental pollutants in a single city, Detroit, allows for interpretation of findings and recommendation development for policymakers within the backdrop of the city’s ongoing need to address and eliminate structural racism and discrimination.

### Public health implications

The environmental exposure most likely to be elevated in redlined vs. non-redlined neighborhoods was close proximity (within 5 km) to a greater number of RMP sites. RMP sites are facilities that produce, use, or transport any of the particularly hazardous or toxic substances (due to their high acute toxicity or flammable/explosive potential, as defined by the Clean Air Act) [45]. These sites are required to develop a RMP for each chemical in preparation for accidental release, and to report the amounts and use of each chemical to the EPA per the Emergency Planning and Community Right-to-Know Act of 1986 [45]. Often these sites are associated with annual releases considered within “allowable” limits for these chemicals and others, but accidental release or risk of accidental release are not reported. As such, historically redlined neighborhoods are the most likely to shoulder the burden from a chemical accident or catastrophe in Detroit, which may pose substantial health implications, many of which would be sudden and unknown to these underserved communities.

**Fig. 4:**
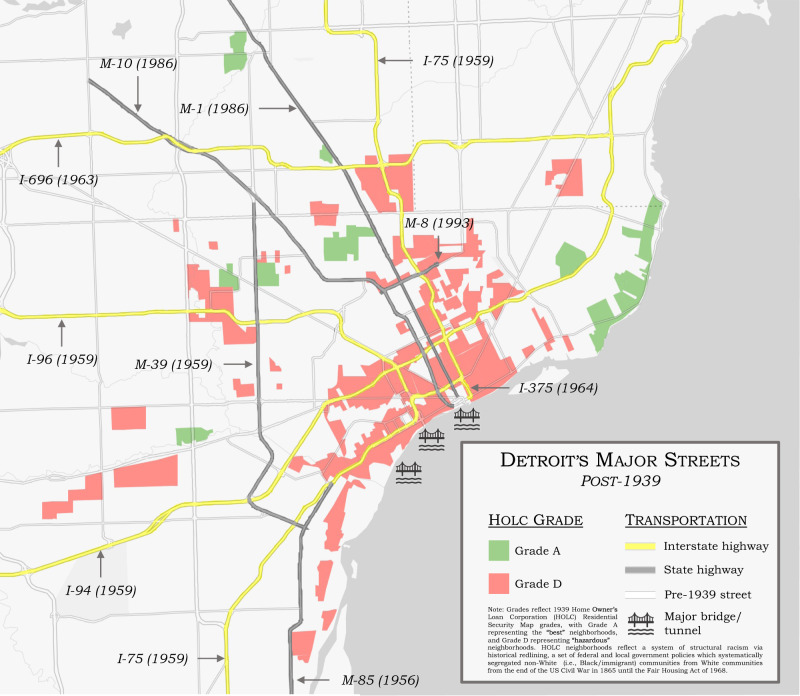
Map of major streets (i.e., interstate and state highways) built post-1939 in Detroit. Green and red shaded areas represent Grade A and D 1939 Home Owners’ Loan Corporation (HOLC) neighborhoods, respectively. Yellow streets represent interstate highways. Gray streets represent state highways. Uncolored streets represent streets constructed pre-1939. Bridges and tunnels (from left to right): Gordie Howe International Bridge (currently under construction); Ambassador Bridge; and Detroit-Windsor Tunnel.

The second most pervasive environmental exposure for redlined neighborhoods was hazardous road noise. Redlined neighborhoods were more likely to have higher proportions of their neighborhoods exposed to hazardous levels of noise. It has been well documented historically that new roads and highways are often build straight through Black and immigrant neighborhoods, as these communities’ political power were often curbed to ensure a lack of resistance to these transportation projects. Figure 4 displays a map of all major streets (i.e., interstate and state highways) constructed in Detroit since 1939 with respect to D-grade and A-grade neighborhoods. While the newly constructed major streets frequently passed straight through redlined neighborhoods, this was not the case for A-grade neighborhoods. In fact, there are only a handful of instances in which the new highways were run even adjacent to A-grade neighborhoods. Furthermore, intersections of interstate highways were frequently placed within redlined communities, highlighting the inclination to gut Black and immigrant minority communities, encircling and confining the residents with noise and air pollution. This history is supported by a 2013 nationwide analysis that found minority and low-income homes situated closer to high traffic volume roads or in an area with higher traffic density [46]. Another study in 2017 found non-white and lower socioeconomic individuals to be disproportionately burdened by noise exposure nationwide [30]. Very recently, this relationship was also shown across some neighborhoods in Detroit [47]. Impacts of noise exposure on hearing are well-characterized; however, noise levels lower than those necessary to harm hearing have been associated with other significant health outcomes, including hypertension, ischemic heart disease, and diabetes [48, 49]. As a result, historically redlined neighborhoods may bear a higher risk of auditory and non-auditory health outcomes of noise exposure.

The third most pervasive environmental exposure for redlined neighborhoods was exposure to diesel PM. Likelihood of a neighborhood being redlined increased substantially when diesel PM was greater than 0.6 µg/m^3^. Although this is below the allowable ambient limit as defined by the EPA for PM_2.5_, the EPA has defined diesel exhaust as a “likely human carcinogen” and has shown it to be associated with many adverse health outcomes, such as respiratory, cardiovascular, and neurological effects [50]. Specially, the California Air Resources Board estimates that 70% of all cancer risk from toxic air can be specifically attributed to diesel engine emissions [51]. The finding in California is consistent with the findings in Detroit, in that the lifetime cancer risk from inhalation of air toxics was the fourth most pervasive environmental exposure for redlined neighborhoods. These items taken together highlight the compounding environmental burden that may lead to substantially higher adverse health risk, specifically cancer, among residents of historically redlined neighborhoods.

Among the most pervasive exposures, three of the four are at least partially associated with traffic, whether that be vehicle routes or the source of vehicle fuel. There have been numerous stories and news articles in and around Detroit about semi-trucks idling in the neighborhoods near the international border to Canada (see articles provided in Appendix C of Supplementary Materials). Currently there are two bridges/tunnels to Canada from the city of Detroit (Fig. 4). Despite years of intense public outcry and a health impact assessment case study identifying elevated levels of asthma and other adverse respiratory and cardiopulmonary outcomes among vulnerable populations [52], a third is currently under construction with a projected completion date of 2024 (see Appendix C). These routes are major thoroughfares for the transportation of goods by semi-trucks and trains, resulting in excessive diesel exhaust, and general air and noise pollution. All of these existing and proposed trans-national routes start in neighborhoods that were redlined (Fig. 4). Their placement is not arbitrary and again highlights the continued environmental harm set in motion by policies established nearly 100 years ago.

Fortunately, some of the harm from environmental racism has increasingly been recognized as of recent. The racially divisive highway I-375 (built in a historically redlined neighborhood; Fig. 4) that once demolished Black neighborhoods is set to be torn down by 2025 (see article 5 provided in Appendix C of the Supplementary Material). Currently, proposed plans show a six-lane boulevard as the preferred alternative. While the boulevard replacement will have some effect on noise and air pollution in the area, the effects are likely to be minimal. A 2020 traffic noise analysis of the I-375 preferred alternative found that existing noise levels in the region range from 66 to 72 dBA (near or above the hazardous threshold for hearing of 70 dBA), and that the preferred alternative plan will not significantly change noise levels [53]. In fact, the preferred alternative plan may increase noise levels by as much as 5.3 dBA [53]. This signifies the need for thoughtful urban planning by policymakers if environmental racism of the past is to be appropriately addressed.

Although not the focus of this paper, one demographic indicator (% without a high school diploma) passed the variable importance threshold in BCT. This is not an entirely surprising finding given that home values impact taxes and taxes fund schools in Michigan. The devaluing of neighborhoods leads to underfunded educational systems and is an injustice itself. Investing in school systems, particularly in redlined neighborhoods may be another avenue, independent of environmental improvements, which may work to undo and right some of the ongoing impacts of historically redlining.

## Conclusions

Our findings suggest that the structural racism and discrimination of historical redlining have led to environmental injustices in Detroit today. Air and noise pollution from transportation sources, particularly from sources of diesel exhaust, may represent primary targets for interventions intended to ameliorate the impacts of historical redlining. Such interventions could include policies (e.g., no idling of diesel trucks and green space requirements for urban planning) and engineering controls (e.g., PM filters for diesel engines, barrier walls around major roadways, electrification of the vehicle fleets, and air filtration units for homes and schools). Future research should examine the role of environmental injustices in contributing to health disparities among cohorts of people in historically redlined cities. Specifically, the findings of this study indicate a plausible biological pathway through which redlining could increase the risk of developing cancer and adverse respiratory health outcomes from air toxics, as well as auditory and non-auditory health outcomes from noise pollution. This warrants further research that examines whether residents living in, or have moved in or out of, historically redlined neighborhoods in Detroit have elevated prevalence or incidence of these adverse health outcomes. However, this research should not delay crucial action from policymakers and advocates in Detroit, and Michigan more broadly.

### Supplementary information

Supplemental material

Reporting Checklist

## Data Availability

All data sources used in this analysis were extracted from publicly available resources.
