# Supplementary material for: Linking environmental injustices in Detroit, MI to institutional racial segregation through historical federal redlining
Source: J Expo Sci Environ Epidemiol. 2022 Dec 21;34(3):389–98. doi: 10.1038/s41370-022-00512-y (PMC11222141; doi:10.1038/s41370-022-00512-y)
Supplement: Supplementary file 1 — Supplemental material [file 41370_2022_512_MOESM1_ESM.docx]

**Supplemental material**

**Linking environmental injustices in Detroit, MI to institutional racial segregation through historical federal redlining**

Abas Shkembi^1^, Lauren M. Smith^1^, Richard L. Neitzel^1^

^1^Department of Environmental Health Sciences, University of Michigan, Ann Arbor, MI, United States

Address correspondence to Abas Shkembi, Department of Environmental Health Sciences, University of Michigan, 1415 Washington Heights, Ann Arbor, Michigan 48109-2029. Email: [ashkembi@umich.edu](mailto:ashkembi@umich.edu).

**Appendix A**

The EPA EJScreen can estimate environmental indicators for any user-defined shape. However, the shapes must be fully enclosed by their outermost sides for proper estimation. Five neighborhoods in the city of Detroit (B1, C16, C89, C107, and D24) were not included in this analysis due to this type of boundary issue; as such, estimation of these neighborhoods’ environmental indicators was not possible given that they would result in even more considerable error than already present in the estimation methods. The figures in Supplemental Table S1 display each neighborhood’s shapefile overlaid onto the EJScreen to demonstrate this phenomenon.

| **Table S1.** |  |  |
| --- | --- | --- |
| **Neighborhood** | **EPA EJScreen screenshot** | **Issue** |
| B1 | 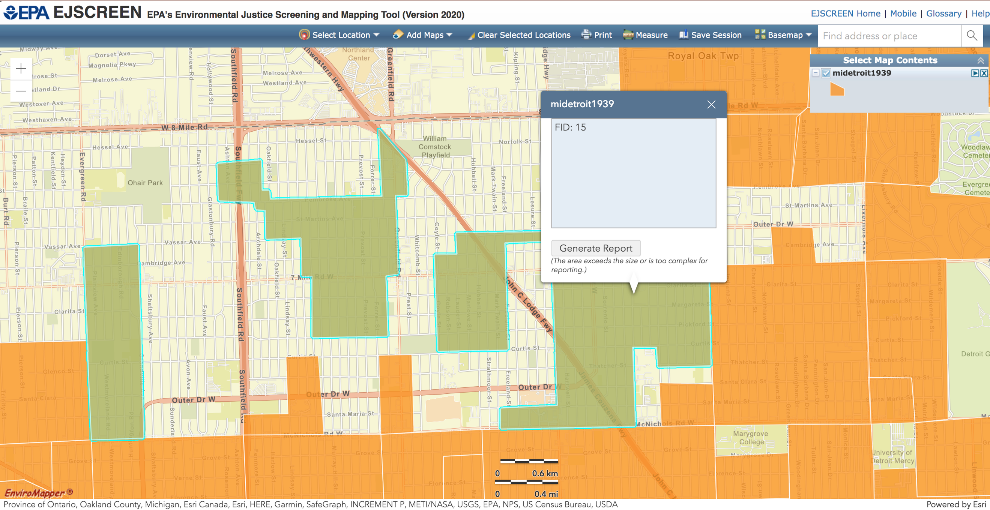 | Three distinct shapes. |
| C16 | 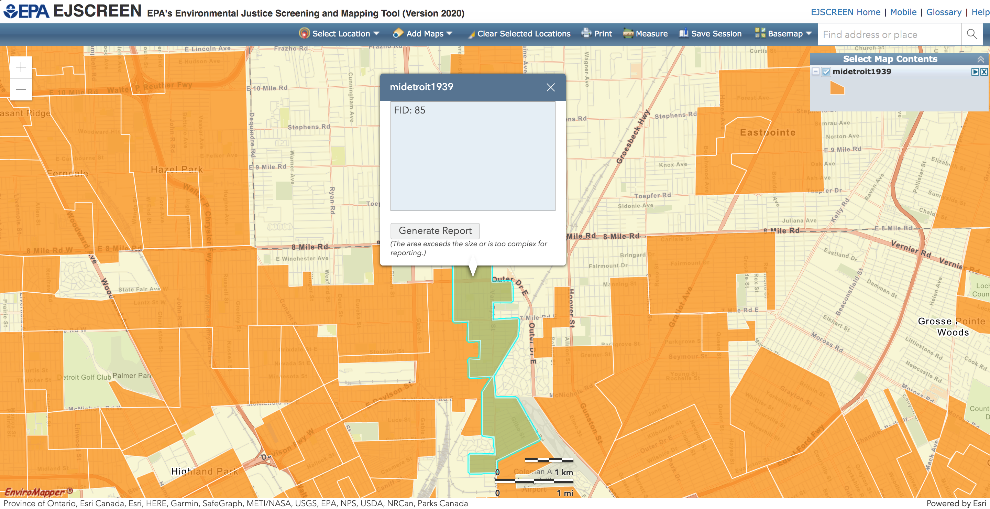 | Two distinct shapes. |
| C89 | 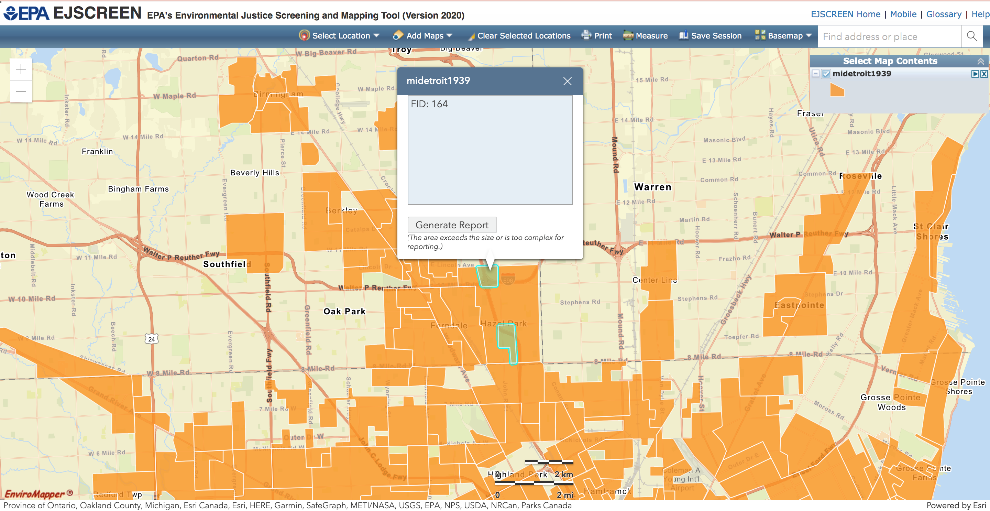 | Two distinct shapes. |
| C107 | 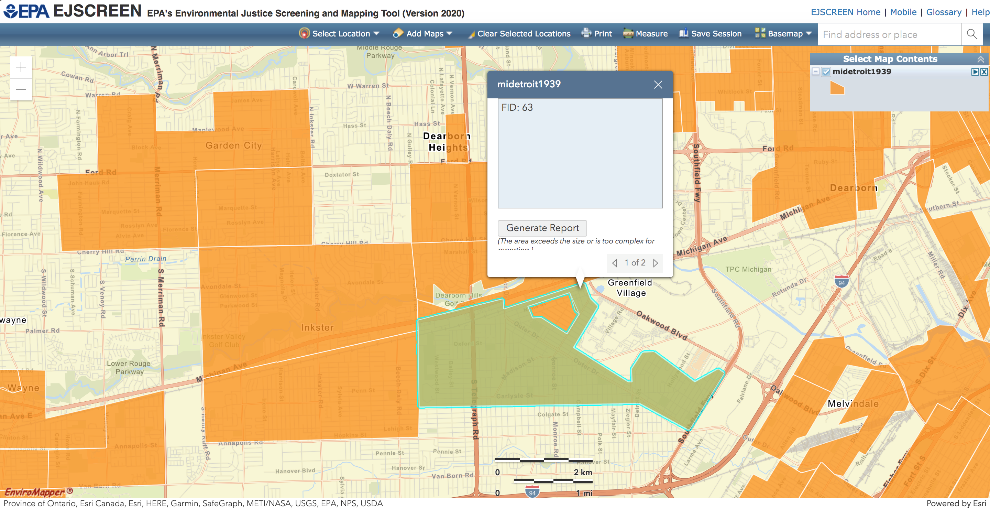 | Area of shape not fully enclosed by outermost sides. |
| D24 | 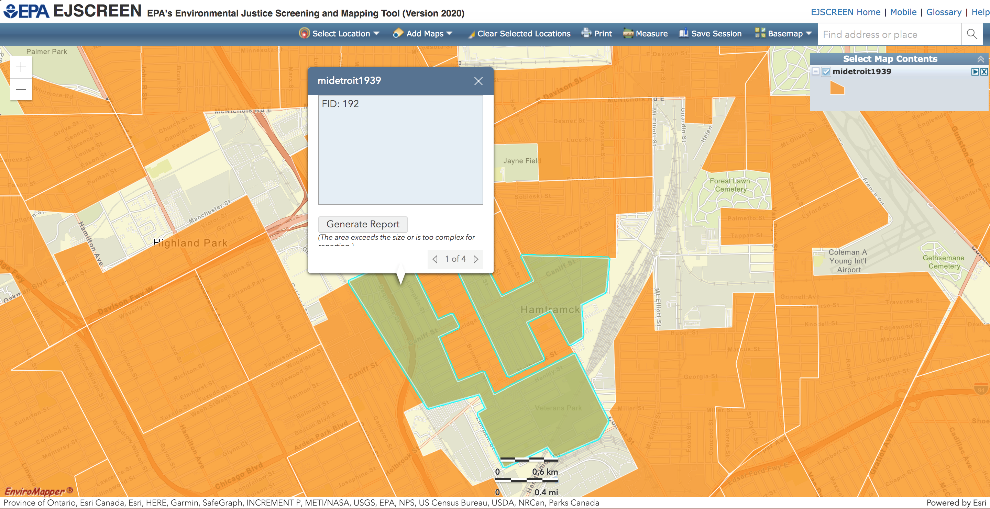 | Two distinct shapes. |

**Appendix B**

**Figure S1.** Depiction of the hazardous noise indicator from neighborhood D49 in Detroit, Michigan (red highlighted). For each HOLC neighborhood, we counted the number of road noise estimates that were >70 dBA (signified by Xs), a level considered hazardous to hearing in a community setting, among all noise measurements (Xs and Os) to determine the percentage of road noise by neighborhood as a “hazardous noise” environmental indicator. Note that a random sample of 20% of road noise estimates are shown in the figure for illustrative purposes.

**Figure S2.** Boxplots of demographic indicators by 1930s HOLC grade. From left to right, top to bottom, percent of population: <5 years old, >64 years old, with no high school (HS) diploma, linguistically isolated, low income, and minority (or person of color). Grade A signifies the “best” neighborhoods; grade B signifies “still desirable” neighborhoods; grade C signifies “definitely declining” neighborhoods; and grade D signifies “hazardous” neighborhoods. Redlined neighborhoods (grade D) are shaded grey; non-redlined neighborhoods (grades A, B, and C) are not shaded. Diamond represents mean.

**Figure S3.** Results of the boosted classification tree analysis using D- and C-graded neighborhoods only. The left panel displays the relative variable importance of environmental and demographic indicators strongly associated with a redlined neighborhood compared to a C-graded neighborhood. Predictors that exceed the dashed line (i.e., randomness threshold of 100/14, or 7.14%) are considered important factors that are most indicative of an area having been redlined. Grey bars are environmental indicators; white bars are demographic indicators. The right panel displays the partial dependency plots for the three environmental indicators and one demographic indicator above the randomness threshold in order of decreasing relative importance (from left to right, top to bottom): proximity to risk management plan sites (count); diesel particulate matter (PM, µg/m^3^); hazardous noise indicator (% road noise exposure >70 dBA); and D_Index_, the average percent of low-income minorities). The black line signifies the smoothed partial dependency plot.

| **Table S2.** Comparison of percent difference in environmental indicator levels between D/A-C graded neighborhoods and D/C graded neighborhoods | | |
| --- | --- | --- |
|  | **Percent difference (95% CI)** | |
| **Environmental Indicator** | **D (n = 61) vs A-C (n = 172)** | **D (n = 61) vs C (n = 119)** |
| RMP sites | 98.7 (48.0, 167) | 70.3 (24.2, 134) |
| Hazardous waste sites | 69.4 (35.8, 111) | 42.5 (14.2, 77.8) |
| Hazardous noise indicator | 65.7 (8.6, 152.8) | 62.4 (3.1, 155.9) |
| Traffic volume | 32.2 (3.3, 69.3) | 24.6 (-4.3, 62.3) |
| Diesel PM | 12.1 (7.2, 17.1) | 9.8 (4.8, 14.8) |
| Air cancer toxics risk | 4.7 (2.9, 6.6) | 3.7 (1.7, 5.8) |
| Respiratory hazard index | 3.9 (2.1, 5.6) | 3.2 (1.5, 4.9) |
| Lead paint indicator | -13.6 (-19.6, -7.6) | -11.9 (-18.7, -5.0) |
| PM_2.5_ | 0.5 (0.0, 1.0) | 0.3 (-0.3, 0.8) |
| Ozone | -0.1 (-0.8, 0.6) | -0.1 (-0.7, 0.6) |
| Superfund sites | -4.9 (-19.6, 12.4) | 0.9 (-13.9, 18.3) |
| Wastewater sites | 40.0 (-69.0, 532) | 46.9 (-69.8, 614) |
| Note. Units of environmental indicators, from top to bottom: proximity to Risk Management Plan (RMP) sites (count), proximity to hazardous waste sites (count), hazardous noise indicator (% road noise exposure >70 dBA), traffic volume (count), diesel particulate matter (PM, µg/m^3^), air cancer toxics risk (lifetime risk in 1 million), respiratory hazard index (ratio – unitless), lead paint indicator (% housing stock pre-1960s), PM_2.5_ (µg/m^3^), ozone (ppb), proximity to Superfund sites (count), and proximity to wastewater sites (count). | | |

**Appendix C**

A set of articles detailing public perceptions on semi-truck idling in neighborhoods near bridges to Canada,^1,2^ as well as articles on the construction of the Gordie Howe International Bridge^3,4^ and plans to tear down highway I-375.^5^

1. CBC News. Idle No More draws hundreds to Ambassador Bridge. *CBC*. https://www.cbc.ca/news/canada/windsor/idle-no-more-draws-hundreds-to-ambassador-bridge-1.1302410. Published January 11, 2013. Accessed January 26, 2022.

2. Perkins T. Reining in the fumes: New efforts aim to get control over idling trucks and buses in Detroit and Michigan. *Planet Detroit*. https://planetdetroit.org/2021/09/reining-in-the-fumes-new-efforts-aim-to-get-control-over-idling-trucks-and-buses-in-detorit-and-michigan/amp/. Published September 30, 2021. Accessed January 26, 2022.

3. Rahal S. Gordie Howe bridge on track to open in 2024 despite construction delays. *The Detroit News*. November 20, 2020.

4. CBC News. 1,000 days of construction: Gordie Howe International Bridge surpasses milestone. *CBC*. https://www.cbc.ca/news/canada/windsor/gordie-howe-bridge-construction-1.6094328. Published July 8, 2021. Accessed January 26, 2022.

5. Associated Press. Buttigieg Awards Grant to Tear Down Divisive Detroit Highway. *US News*. https://www.usnews.com/news/business/articles/2022-09-15/buttigieg-awards-big-fed-grant-to-dismantle-racist-highway#:~:text=After%20years%20of%20planning%20dating,with%20construction%20finished%20by%202028. Published September 15, 2022. Accessed November 6, 2022.
